# High Seroprevalence of Rift Valley Fever and Evidence for Endemic Circulation in Mbeya Region, Tanzania, in a Cross-Sectional Study

**DOI:** 10.1371/journal.pntd.0001557

**Published:** 2012-03-27

**Authors:** Norbert Heinrich, Elmar Saathoff, Nina Weller, Petra Clowes, Inge Kroidl, Elias Ntinginya, Harun Machibya, Leonard Maboko, Thomas Löscher, Gerhard Dobler, Michael Hoelscher

**Affiliations:** 1 Division of Infectious Diseases, Medical Center of the University of Munich, Munich, Germany; 2 German Centre for Infection Research (DZIF), University of Munich, Munich, Germany; 3 NIMR-Mbeya Medical Research Programme, Mbeya, Tanzania; 4 Mbeya Regional Medical Office, Ministry of Health, Mbeya, Tanzania; 5 Bundeswehr Institute of Microbiology, Munich, Germany; Centers for Disease Control and Prevention, United States of America

## Abstract

**Background:**

The Rift Valley fever virus (RVFV) is an arthropod-borne phlebovirus. RVFV mostly causes outbreaks among domestic ruminants with a major economic impact. Human infections are associated with these events, with a fatality rate of 0.5–2%. Since the virus is able to use many mosquito species of temperate climates as vectors, it has a high potential to spread to outside Africa.

**Methodology/Principal Findings:**

We conducted a stratified, cross-sectional sero-prevalence survey in 1228 participants from Mbeya region, southwestern Tanzania. Samples were selected from 17,872 persons who took part in a cohort study in 2007 and 2008. RVFV IgG status was determined by indirect immunofluorescence. Possible risk factors were analyzed using uni- and multi-variable Poisson regression models. We found a unique local maximum of RVFV IgG prevalence of 29.3% in a study site close to Lake Malawi (N = 150). The overall seroprevalence was 5.2%. Seropositivity was significantly associated with higher age, lower socio-economic status, ownership of cattle and decreased with distance to Lake Malawi. A high vegetation density, higher minimum and lower maximum temperatures were found to be associated with RVFV IgG positivity. Altitude of residence, especially on a small scale in the high-prevalence area was strongly correlated (PR 0.87 per meter, 95% CI = 0.80–0.94). Abundant surface water collections are present in the lower areas of the high-prevalence site. RVF has not been diagnosed clinically, nor an outbreak detected in the high-prevalence area.

**Conclusions:**

RVFV is probably circulating endemically in the region. The presence of cattle, dense vegetation and temperate conditions favour mosquito propagation and virus replication in the vector and seem to play major roles in virus transmission and circulation. The environmental risk-factors that we identified could serve to more exactly determine areas at risk for RVFV endemicity.

## Author Summary

We describe a high seropositivity rate for Rift Valley fever virus, in up to 29.3% of tested individuals from the shore of Lake Malawi in southwestern Tanzania, and much lower rates from areas distant to the lake. Rift Valley fever disease or outbreaks have not been observed there in the past, which suggests that the virus is circulating under locally favorable conditions and is either a non-pathogenic strain, or that occasional occurrence of disease is missed. We were able to identify a low socio-economic status and cattle ownership as possible socio-economic risk factors for an individual to be seropositive. Environmental risk factors associated with seropositivity include dense vegetation, and ambient land surface temperatures which may be important for breeding success of the mosquitoes which transmit Rift Valley fever, and for efficient multiplication of the virus in the mosquito. Low elevation of the home, and proximity to Lake Malawi probably lead to abundant surface water collections, which serve as breeding places for mosquitoes. These findings will inform patient care in the areas close to Lake Malawi, and may help to design models which predict low-level virus circulation.

## Introduction

The Rift Valley fever virus (RVFV), a member of the genus Phlebovirus in the family Bunyaviridae, was first isolated in 1930 during an outbreak in Kenya. Rift Valley fever (RVF) occurs endemically and epidemically in most parts of sub-Saharan Africa and epidemically in Egypt, Madagascar and the Comoros. In 2001 it was detected for the first time outside of Africa during an outbreak in Yemen and Saudi-Arabia [1], [2], [3], [4], [5].

The disease is mostly apparent in epizootic events with large numbers of sick cattle, and a high abortion rate in pregnant animals (“abortion storm”), with adverse economic consequences for cattle herders, including bans on animal trade [4]. Transmission to humans is common during such events. In the majority of cases, human infection is oligo- or asymptomatic, but may cause hepatitis, hemorrhagic fever, encephalitis and retinitis, with fatality rates of 0.5 to 2%, and permanent vision impairments after retinitis [4].

Contrary to the assumption of virus persistence and inactivity between outbreaks, some evidence for inter-epidemic circulation of RVFV has been reported from the Senegal and from northern Kenya, using a serology approach to detect antibodies in samples from children born after the last reported outbreak [6], [7].

The most important vectors for RVFV are *Aedes* and *Culex* mosquitoes. However, RVFV has also been isolated from *Anopheles spp*, *Simulium* blackflies, sand flies and *Amblyomma* ticks [2], [4], [8], which may represent remnants of a blood meal rather than the ability to transmit the pathogen. Direct transmission through infectious body fluids is of relevance mainly during epizootic/epidemic events [5], [9]. As many competent vector species occur outside Africa, a high potential for further geographical spread is attributed to the virus, and RVF is classified as an emerging disease [4], [10].

RVF outbreaks are known to occur predominantly after unusual flooding events. *Aedes* mosquito species are seen as vectors and reservoir, since their transovarially infected eggs withstand desiccation and larvae hatch when in contact with water [6], [11]. Transovarial transmission is assumed as mechanism of virus persistence between epizootic events.

After flooding, the *Aedes* mosquito populations will multiply in the persisting water collections, and develop into infectious adult mosquitoes. The RVFV may amplify in wild and domestic ungulates and may reach epizootic and epidemic dimensions [8]. The presumed link between extraordinary flooding events and RVF outbreaks was validated, among others, by a successful prediction of the 2007 outbreak in Somalia, Kenya and northern Tanzania, using climate modelling [12].

A number of variables associated with higher likelihood for RVFV Immunoglobulin G (RVFV IgG) positivity have been identified. Among them are the proximity to perennial surface water bodies and proximity to ruminants [7], [13].

Here we report a cross-sectional seroprevalence study that used samples from 1228 participants collected during a cohort study (EMINI) from the Mbeya region in Southwestern Tanzania, an area from which no RVF disease activity has been reported previously. The objective was to assess any RVFV circulation that had possibly remained undetected, and to describe infection patterns and factors associated with seropositivity.

## Methods

### Ethics statement

Both EMINI and this substudy were approved by Mbeya Medical Research and Ethics Committee, Tanzanian National Institute for Medical Research – Medical Research Coordinating Committee, as well as by the Ethical Commission of University of Munich. Each EMINI participant had provided written informed consent before enrolment. Parents consented for participation of their children.

### Study population

**Figure 1:**
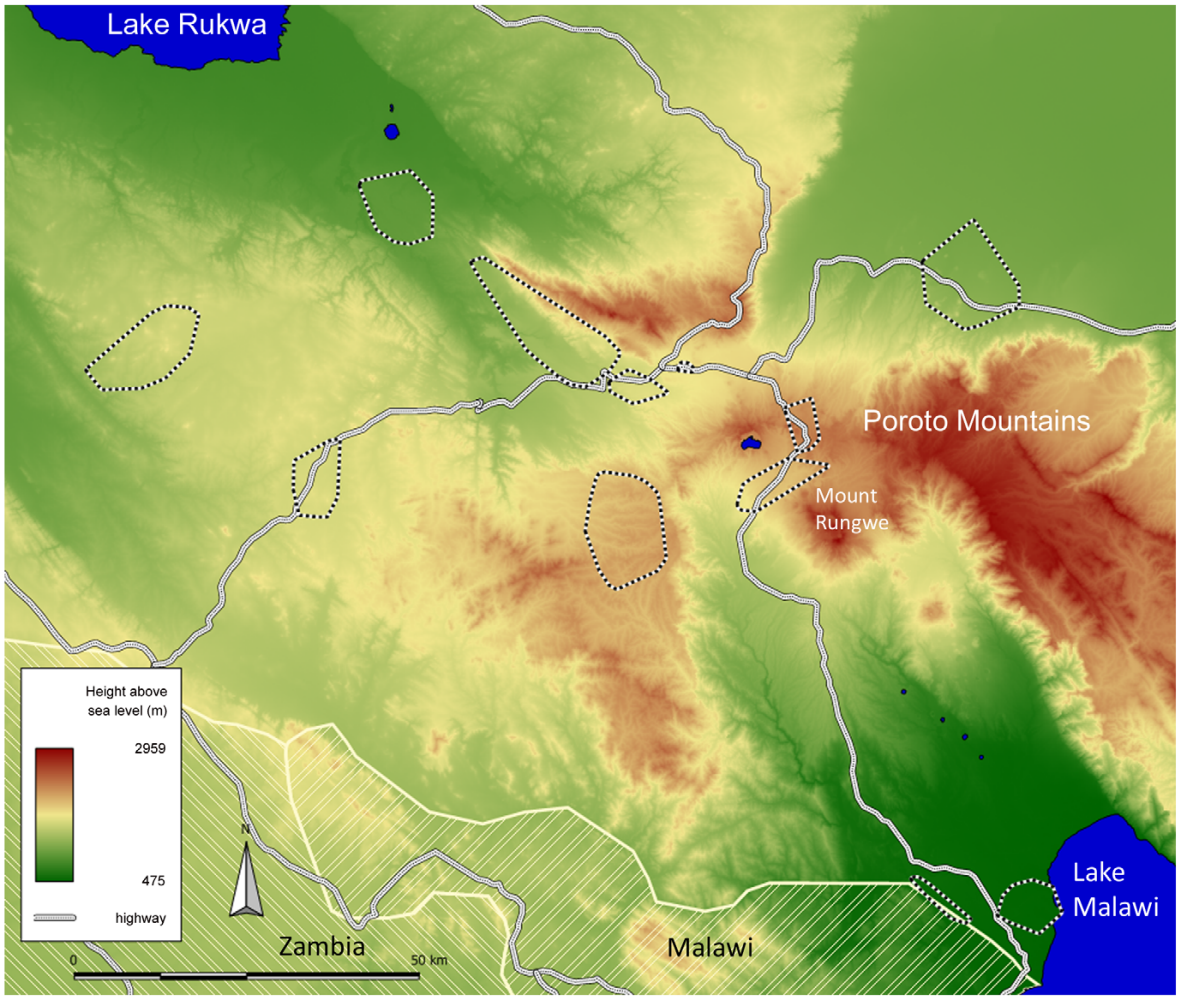
Location and Elevation of the Study Sites. A geographical map of the study area and sites.

Data and samples for this study were collected between June 2007 and June 2008 during the second annual survey of the EMINI (Evaluating and Monitoring the Impact of New Interventions) cohort study. Before the start of EMINI, a census of the complete population had been conducted in nine geographically distinct sites of the Mbeya Region in Southwestern Tanzania, which had been selected to represent a wide variety of environmental and infrastructural settings, including urban and rural sites, different proximity to main roads, elevation above sea-level etc (Figure 1). During the census we collected basic information regarding the households and their inhabitants, and recorded all household positions, using handheld GPS receivers. Ten percent of the census households and all their inhabitants were chosen by geographically stratified random selection to participate in the 5-year longitudinal EMINI cohort study, resulting in a representative sample of the population in the nine study sites. Every year, each participating household was visited to conduct structured interviews and to collect blood and other specimen from all household members. Blood samples were cryo-preserved after cells were separated from serum.

For this substudy, we stratified the 17,872 participants, who had provided a blood sample in the second EMINI survey, by age, gender, altitude of residence and ownership of domestic animals (mammals), to be able to assess factors of interest that were identified in the literature but might have been underrepresented in the general population. We employed disproportionate random sampling with equal participant numbers for each stratum to identify 1228 samples from participants above the age of 5 years to be tested for RVFV IgG.

### Socio-economic status (SES)

During the annual EMINI visits, we conducted interviews with the head of each household regarding the socio-economical and infrastructural setting in and around the household. With this information we constructed an SES score that characterizes the socio-economic situation of each household, employing a modification of a method originally proposed by Filmer & Pritchett that uses principal component analysis and has been widely applied to assess wealth and poverty in developing countries [14], [15], [16]. The score included the following information: Availability of different items in the household (clock or watch, radio, television, mobile telephone, refrigerator, hand cart, bicycle, motor cycle, car, savings account); sources of energy and drinking water; materials used to build the main house; number of persons per room in the household and availability and type of latrine used.

### Environmental data

Population- and livestock-densities were calculated using data and household positions collected during the initial population census.

Elevation data were retrieved from the NASA Shuttle Radar Topography Mission (SRTM) global digital elevation model, version 2.1 [17], [18].

Land surface temperature (LST) and vegetation cover (EVI = enhanced vegetation index) data were retrieved from NASA's Moderate-resolution Imaging Spectroradiometer (MODIS) Terra mission which “are distributed by the Land Processes Distributed Active Archive Center (LP DAAC), located at the U.S. Geological Survey (USGS) Earth Resources Observation and Science (EROS) Center (lpdaac.usgs.gov).” [19]. LST data (Version MOD11A2) have 8 days temporal and ∼1 km spatial resolution , EVI data (Version MOD13Q1) have 16 days temporal and 250 m spatial resolution. Both, LST and EVI data were processed in the following way to produce long-term averages: After download via FTP, data surfaces for every 8 day period for the years 2000 to 2008 (LST) and every 16 days period for the year 2007 (EVI) were reprojected to Universal Transverse Mercator projection (zone 36 South) using the MODIS reprojection tool (MRT) [20] and imported into Idrisi GIS software (version 32, Clark Labs, Worcester, MA, USA). In Idrisi, 8 year averages of annual average and maximum day-LST and average and minimum night-LST and 2007 EVI averages were calculated for each pixel utilising only those pixels that were “good quality” according to the quality assessment layers that are distributed together with the actual data. Then LST was converted to degrees Celsius and EVI was converted back to its native range between −1 and +1.

All above environmental data were then combined with the houshold position data in a GIS database using Manifold System 8.0 Professional Edition (Manifold Net Ltd, Carson City, NV). Population-, household-, and livestock-densities, LST, EVI, and elevation data were averaged for a buffer area within 1000 meter radius around each household in order to characterize the ecological situation around the household. This approach was preferred to using the respective spot values at the household position, because spot data are more prone to random error than averages for a wider area.

### Serology

Anti-RVFV IgG was detected by indirect immunofluoerescence assay (IIFA), following a methodology adapted from Swanepoel [21]. Each serum sample was screened for the presence of anti-RVFV IgG, using a commercial biochip with a mixture of infected and non-infected Vero E6 cells on one field (positive field) and non-infected Vero E6 cells on a negative control field (Euroimmun, Lübeck, Germany).

Sensitivity and specificity of the IIFA test were tested using 20 negative sera from German blood donors and five sera positive for IgG against Sandfly Toscana virus, Sandfly Naples virus, Sandfly Sicilian virus, Puumala virus, Tahyna virus and Bunyamwera virus. No cross reactivities with other members of the genus Phlebovirus or the viruses of other genera of the family Bunyaviridae were detected.

Serum samples were screened in a dilution of 1∶10, using standard procedures for IIFA. A rabbit anti-human IgG FITC-labelled antibody (DAKO, Hamburg, Germany) was used as conjugate. A sample was classified as positive if a typical fine granular cytoplasmatic fluorescence in some groups of cells on the positive field of the biochip was detected, with no detectable fluorescing cytoplasmatic signal in the negative field. Each sample was independently assessed by two experienced observers. Results were compared and re-tested if discrepant. A part of the positive sera was re-tested by titration, and all tested sera were found to have IgG titres between 1∶20 and 1∶640.

### Data analysis

Stata statistics software (version 11, Statacorp, College Station, TX, USA) was used for all statistical analyses, maps were produced in Manifold System 8.0 Professional Edition (Manifold Net Ltd, Carson City, NV).

After exploratory data analysis, it became clear that RVFV seroprevalence in Bujonde-Kajunjumele (BK) subsite was much higher than in all other study locations. We therefore decided to first analyse data for BK separately before trying to develop models including the data for all sites.

Since none of the continuous variables that we examined was normally distributed according to the Shapiro-Wilk and Shapiro-Francia tests for normality, the median and interquartile range (instead of mean and standard deviation) of these variables are reported to characterize the study area and population in BK and in all other sites. This is also the reason why the non-parametric Wilcoxon ranksum test was used to assess differences between BK and all other sites regarding continuous variables. Differences between sites regarding binary variables (RVF seropositivity, gender and cattle ownership) were assessed by chi square testing. The association of binary RVFV IgG status with possible risk factors was examined using uni- and multi-variable poisson regression models with robust variance estimates adjusted for within household clustering [22], [23]. Uni-variable regression models were used to identify possible risk-factors for inclusion into the multi-variable model for this site. Variables with a p-value<0.2 in uni-variable regression and other variables that did not fulfill this criterion, but where an association with RVFV IgG seemed likely due to biological reasons (gender, and all variables related to the presence of ruminants), were further evaluated in multi-variable regression models and were retained in the final multi-variable model if their p-value was <0.1. Because most variables characterizing the natural environment (LST, vegetation, elevation and distance to Lake Malawi) showed strong collinearity, they were not included into the same model but entered one by one into models adjusted for the other variables that were included into the final model.

Once the final multi-variable model for BK site was identified, we used the same approach to identify a multi-variable model where data for all sites including BK were pooled. Prevalence ratios (PR) and 95% confidence intervals for covariates mentioned in the text refer to multi-variate analysis within BK site, if not mentioned otherwise.

## Results

### Site characteristics and seroprevalence

Of the 1228 analyzed sera, 5.2% (64 sera) were positive for RVFV IgG. This translates into an estimated overall population prevalence of 3.1% when extrapolated from our stratified sample to the underlying population of the 9 study sites.

**Figure 2:**
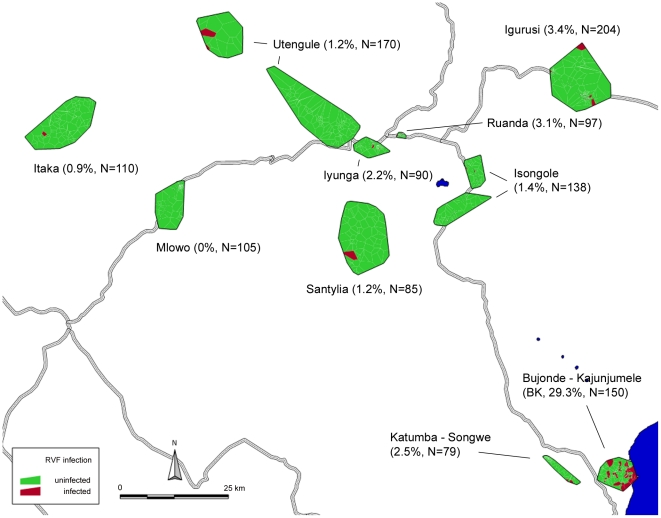
Location of Households with IgG-positive Participants in the entire Study Area. Location of households displayed in Voronoi polygons, with every polygon representing one household.

**Figure 3:**
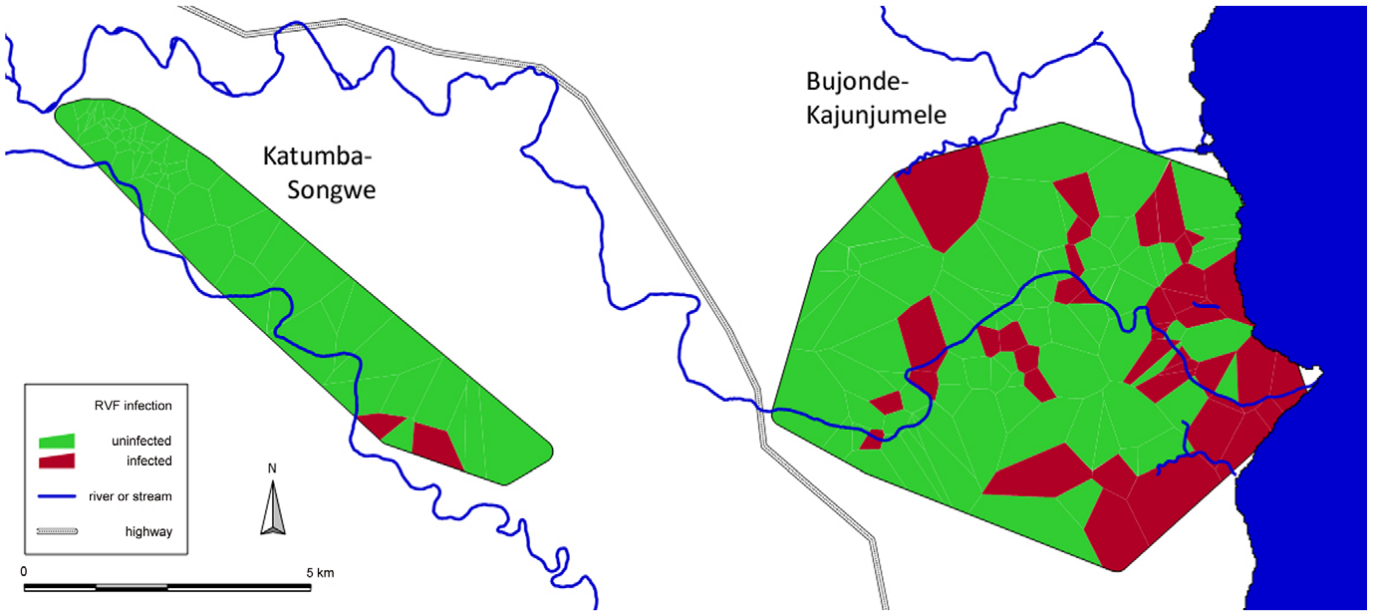
Location of households with RVF IgG-positive participants in Katumba-Songwe and Bujonde-Kajunjumele (BK) sites. Location of households with RVF IgG-positive participants displayed as Voronoi polygons.

We found a unique local maximum of 29.3% (95% confidence interval (CI) 22.2–37.3) seroprevalence in Bujonde-Kajunjumele (BK), a subsite of the Kyela site, which is situated close to Lake Malawi. The prevalence in the other sites ranged from 0.0% to 3.4% (table 1, Figure 2, 3).

**Table 1:** Characteristics of Bujonde-Kajunjumele site (N = 150) and all other sites (N = 1078).

	BK-siteMedian (IQR) or % (N)		All other sitesMedian (IQR) or % (N)		p^a^
RVF IgG positive	29.3%	(44)	1.9%	(20)	<0.001
Female gender	55%	(82)	55%	(590)^b^	0.885
Age (years)	32.1	(17.8 to 53.2)	34.2	(16.9 to 51.9)	0.684
SES score	−0.81	(−1.19 to −0.42)	−0.06	(−0.54 to 0.55)	<0.001
Cattle owned	49%	(74)	28%	(307)	<0.001
Elevation (meters)	484	(481 to 487)	1570	(1207 to 1714)	<0.001
Vegetation Density (EVI*10)	3.83	(3.60 to 4.05)	2.87	(2.56 to 3.27)	<0.001
Max. LST (°C)	43.1	(39.5 to 45.4)	45.7	(42.9 to 46.5)	<0.001
Average Day LST (°C)	30.9	(29.3 to 31.8)	32.5	(30.2 to 33.2)	<0.001
Min. LST (°C)	14.9	(13.5 to 15.7)	11.0	(7.3 to 11.9)	<0.001
Cattle Density (cows/km^2^)	165	(143 to 190)	68	(34 to 186)	<0.001
Distance to Lake Malawi (km, BK only)	2.9	(1.4 to 5.2)			

BK = Bujonde-Kajunjumele; IQR = interquartile range; SES = socio-economic status; EVI = enhanced vegetation index; LST = land surface temperature.

a p-value of chi square test (for binary variables) or Wilcoxon Ranksum test (for continuous variables) for difference between BK and all other sites.

b gender unclear for 11 participants.

We thus decided to analyze covariates within the high-prevalence setting of BK site, and to compare BK to the low-prevalence sites, in order to better understand possible causes for this marked difference.

With an altitude range of 479 to 492 meters, BK is the lowest of our study sites, while the other sites range from 499 m to 2316 m (table 1, Figure 1). The two Kyela subsites BK and Katumba-Songwe are the only sites south of the Poroto mountain range, and receive the highest amount of annual rainfall (1956 and 2292 mm, respectively), whereas the average across all sites is 1473 mm.

**Figure 4:**
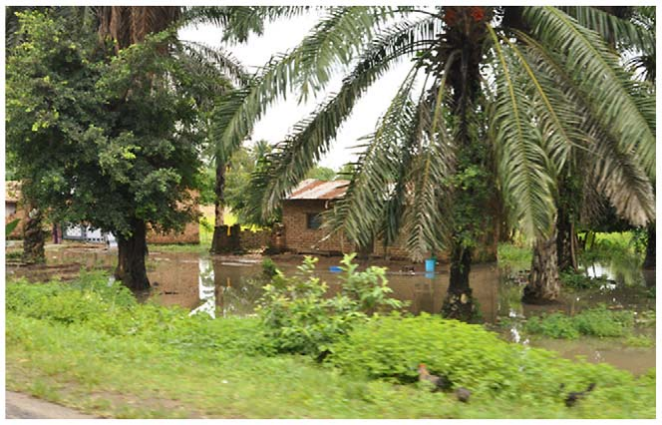
Surface Water Collections in Bujonde-Kajunjumele Site. Surface water collections situated close to Lake Malawi. At the end of the rainy season in April, the high prevalence area close to Lake Malawi, with elevations barely above the Kiwira river water level, is characterized by abundant waterlogging, with surface water between homesteads.

Further characteristics of BK site, compared to all other sites, are listed in table 1. Of special relevance are denser vegetation, lower temperature variability (higher minimum and lower maximum land surface temperatures), higher cattle density and more frequent ownership of cattle, which is presumed to be the main animal host of RVFV. During the rainy season, wide areas close to Lake Malawi are flooded, especially where the terrain is marshy and barely above the Kiwira river's water level (Figure 4).

### Analysis of potential risk-factors

**Figure 5:**
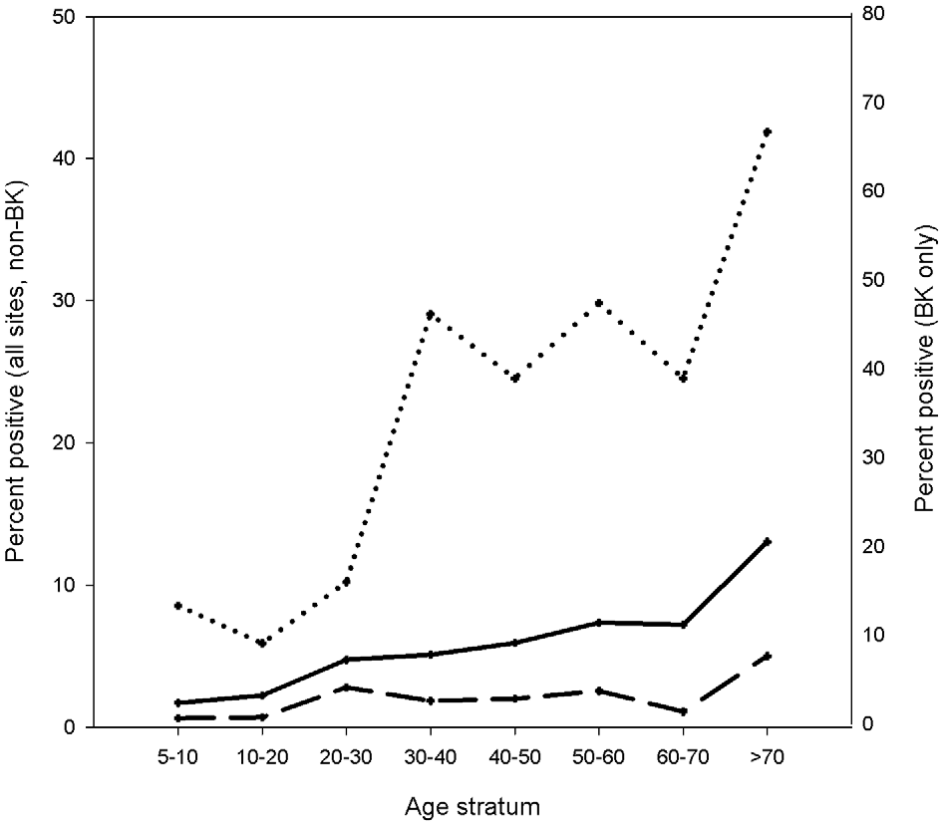
Seropositivity of RVFV IgG by Age. Dotted line: percent positives in BK site, N = 150. Solid line: percent positives in all sites including BK, N = 1228. Dashed line: percent positives in all sites other than BK, N = 1078. Please note the different scales for BK (right axis) and for all sites and non BK sites (left axis).

As demonstrated in Figure 5, RVFV IgG prevalence rises with age in our study population. This is in agreement with the poisson regression results for BK (table 2; prevalence ratio (PR) 1.02 per year of age, 95% CI 1.01–1.03), and for the pooled results from all sites, where age is significantly associated with rising RVFV IgG prevalences, both in uni- and in multi-variable regression models (table 3; PR 1.02 per year of age, 95% CI 1.01–1.03). Increasing socio-economic status is associated with decreasing RVFV IgG prevalences (BK site: PR 0.60 per unit, 95% CI 0.40–0.90), whereas gender appears not to influence RVFV IgG prevalence in the study population (uni-variable PR for male gender as compared to female in BK, 1.0, 95% CI 0.61–1.65).

**Table 2:** Socio-economic and environmental factors and association with RVFV IgG^a^ in BK.

Covariate		univariable			multivariable^b^ ^d^		
	stratum	PR	(95%CI)	p	PR	(95%CI)	p
Gender^b^							
	female^c^	1					
	male	1.00	(0.61 to 1.65)	0.985			
Age							
	per year	1.02	(1.01 to 1.04)	<0.001	1.02	(1.01 to 1.03)	<0.001
SES score							
	per unit	0.53	(0.34 to 0.84)	0.006	0.60	(0.40 to 0.90)	0.014
Cattle owned							
	no^c^	1			1		
	yes	1.23	(0.74 to 2.05)	0.421	1.81	(1.15 to 2.85)	0.010
Cattle per sqkm^b^							
	per 100	0.84	(0.48 to 1.45)	0.522			
Vegetation (EVI)							
	per unit	3.98	(1.84 to 8.61)	<0.001	2.99	(1.34 to 6.65)	0.007
					Results for other environmental variables when included into the above model instead of vegetation:		
Dist. to Lake Malawi^d^							
	per km	0.74	(0.64 to 0.86)	<0.001	0.79	(0.69 to 0.90)	<0.001
Elevation^d^							
	per m	0.84	(0.77 to 0.93)	<0.001	0.87	(0.80 to 0.94)	0.001
LST maximum^d^							
	per °C	0.84	(0.77 to 0.92)	<0.001	0.87	(0.81 to 0.94)	<0.001
LST average day^d^							
	per °C	0.67	(0.56 to 0.80)	<0.001	0.73	(0.61 to 0.86)	<0.001
LST average night^d^							
	per °C	3.82	(1.28 to 11.45)	0.017	2.51	(0.94 to 6.70)	0.066
LST minimum^d^							
	per °C	1.18	(0.94 to 1.49)	0.149	1.07	(0.88 to 1.31)	0.473

N for BK site = 150.

PR = prevalence ratio; SES = socio-economic status; skm = square kilometre; EVI = enhanced vegetation index; LST = land surface temperature.

a results of uni- and multivariable poisson regression with robust variance estimates adjusted for clustering within household.

b gender and cattle per skm were not included into multivariable model due to lack of significance.

c reference stratum.

d to avoid collinearity problems, the environmental variables (vegetation, distance to lake, elevation and the four LST variables) were entered separately into models adjusted for age, SES, and cattle ownership. Multivariable results for these three adjustment variables are those for the model that included vegetation.

**Table 3:** Socio-economic and environmental factors and association with RVFV IgG^a^ in all sites, including BK.

Covariate		univariable			multivariable^b^		
	stratum	PR	(95%CI)	p	PR	(95%CI)	p
Gender^b^							
	Female^c^	1					
	male	1.12	(0.69 to 1.82)	0.643			
Age							
	per year	1.02	(1.01 to 1.04)	<0.001	1.02	(1.01 to 1.03)	<0.001
SES score							
	per unit	0.31	(0.20 to 0.48)	<0.001	0.51	(0.33 to 0.78)	0.002
Cattle owned							
	no^c^	1			1		
	yes	1.73	(1.05 to 2.86)	0.033	1.76	(1.15 to 2.71)	0.010
Cattle per sqkm							
	per 100	1.67	(1.39 to 2.00)	<0.001	2.06	(1.64 to 2.59)	<0.001
Vegetation (EVI)							
	per unit	6.31	(3.68 to 10.81)	<0.001	2.94	(1.87 to 4.63)	<0.001
					Results for other environmental variables when included into the above model instead of vegetation:		
Elevation^d^							
	per 100 m	0.79	(0.74 to 0.84)	<0.001	0.85	(0.79 to 0.90)	<0.001
LST maximum^d^							
	per °C	0.88	(0.83 to 0.92)	<0.001	0.87	(0.83 to 0.92)	<0.001
LST average day^d^							
	per °C	0.88	(0.82 to 0.94)	<0.001	0.83	(0.76 to 0.92)	<0.001
LST average night^d^							
	per °C	1.46	(1.32 to 1.63)	<0.001	1.31	(1.81 to 1.46)	<0.001
LST minimum^d^							
	per °C	1.45	(1.18 to 1.79)	<0.001	1.21	(0.99 to 1.47)	0.063

N for all sites = 1228.

PR = prevalence ratio; SES = socio-economic status; skm = square kilometre; EVI = enhanced vegetation index; LST = land surface temperature.

a results of uni- and multivariable poisson regression with robust varicance estimates adjusted for clustering within household.

b gender was not included into multivariable model due to lack of significance not included into multivariable model due to lack of significance.

c reference stratum.

d to avoid collinearity problems, the environmental variables (vegetation, elevation and the four LST variables) were entered separately into models adjusted for age, SES, cattle ownership and cattle density. Multivariable results for these four adjustment variables are those for the model that included vegetation.

According to the multi-variable models, cattle ownership is significantly associated with RVFV seroprevalence, both in BK and in all sites (PR 1.81, 95% CI 1.15–2.85 for BK; PR 1.76, 95% CI 1.15–2.71 for all sites), although it's uni-variable association in BK is far from significant. Cattle density per square kilometer is a significant prognostic factor in all sites (PR 2.06 per 100/skm, 95% CI 1.64 to 2.59, multi-variable model), including BK, where mean cattle density is higher than in the other sites.

Due to collinearity between the examined environmental variables, these could not be simultaneously included into one model, but were entered one at a time into multi-variable models that were adjusted for age, SES, cattle ownership, and – for the all-sites pooled model – cattle density. Of these environmental variables, vegetation density (EVI) results in the model with the best fit, both in BK and in the pooled analysis. However, most other environmental factors are also strongly associated with RVFV IgG prevalence. It is noteworthy though, that maximum and average land surface temperature (LST) during the day have significant negative associations (PR 0.87 per °C, 95% CI 0.81–0.94 for max. LST; PR 0.73, 95% CI 0.61–0.86 for average day LST, both multi-variable for BK), whereas average LST during the night has a positive association with RVFV seroprevalence (PR 2.51 per °C, 95% CI 0.94–6.70). Minimum LST was less strongly associated than the other LST variables in the pooled analysis and unrelated to RVFV IgG prevalence in BK site.

Other factors that we examined (population density and the ownership of livestock other than cattle) do not show any strong associations with RVFV infection in our study population (data not shown).

### Association with other mosquito-borne and water-borne infections

Within the EMINI study population our group also collected data on chikungunya virus IgG, *P. falciparum* malaria (ICT Malaria P.f./P.v. ICT Diagnostics, Cape Town, South Africa) and presence of *W. bancrofti* filarial antigen (TropBio® Og4C3 serum ELISA, Townsville, Australia). We found that on a household level, RVFV IgG positivity was strongly associated with chikungunya virus IgG in BK site and in all other sites (PR = 4.3; 95% CI 2.3–8.1; PR = 5.3, 95% CI 2.1–13.5, respectively), and with filarial antigen (PR = 2.2; 95% CI 1.3–3.7) and P.falciparum malaria (PR = 4.2, 95% CI 3.3–5.5) in BK. No association was found with *Schistosoma haematobium* infection in BK, nor in the other sites.

## Discussion

The presented analyses identify several socio-demographic and environmental factors that are strongly associated with RVFV seropositivity in our study population. As prevalences in all sites apart from BK are relatively low, we were unsure whether the few cases in the non-BK sites were autochthonous or imported cases and whether an overall analysis of all sites would really yield credible results. It is therefore reassuring that the results of the BK only analysis and those for all sites are similar.

### Socio-economic variables

Figure 4 shows that RVFV seroprevalence increases with age, which is in line with the regression results for BK and for all sites. This suggests an endemic circulation of RVFV in our study area, rather than a single outbreak event as reason for the detected seroprevalence.

The inverse association of SES with RVFV IgG means that more affluent people are at lower risk of infection. This has been described for many different infectious diseases in a wide range of settings. Importantly, cattle ownership was not used for SES calculation, as it is a direct risk factor.

### Environmental variables

Despite the strong associations that we found for age and SES, most of the examined environmental variables were still significantly associated with RVFV IgG prevalence, when adjusted for possible socio-economic confounding in the multivariable models showing that their association with RVF is independent.

Our findings that cattle ownership and density of cattle in the area are important factors for RVFV seropositivity were to be expected, since ruminants are the main animal host of RVFV [4]. Cattle owners in BK have the habit of tethering their animals on the doorsteps of their houses at night for fear of theft, providing an animal reservoir of RVFV in proximity of humans, and reportedly increasing the number of *Culex* mosquitoes in the house [24].

Some previously described risk factors for RVF were confirmed in our study: dense vegetation and proximity to perennial water bodies were found associated with RVFV seropositivity in ruminant herds in the Senegal and in humans in Gabon [13], [25]. These and other risk factors seem to make the BK site uniquely favorable for human and animal RVFV infection.

In BK, only the low-lying areas close to Lake Malawi are subjected to regular flooding during the rainy season, whereas areas further away from the lake and at slightly higher elevation are not flooded. This phenomenon provides abundant mosquito breeding places in low-lying areas, and is a likely reason for the strong negative association of altitude with RVFV seropositivity, that – in BK site – is already visible on a per meter scale, and for the association with distance from the lake. Frequent waterlogging has lead to large areas of BK site being used for wetland rice cultivation. One report from the 2006–2007 outbreak in Kenya found that soils that retain water were more frequently found in RVF-affected areas than in other areas [26].

Over the entire study area with an altitude range of 479 to 2313 m, it seems obvious that higher elevation negatively affects mosquito breeding and survival.

The association of RVFV seropositivity with other mosquito-borne diseases transmitted by *Anopheles*, *Aedes* and *Culex* species is in agreement with above considerations regarding the role of mosquito favorable habitats as an important factor contributing to RVF prevalence in BK site. However, in our study, RVFV seropositivity is not associated with *S. haematobium*, a water-borne disease. *Bulinus* snails, the intermediate hosts of *S. haematobium*, require permanent water-bodies [27]. Thus, RVFV infection in BK does not seem to depend on proximity to Lake Malawi itself, which is a reservoir for *S. haematobium*, but is more likely a consequence of the seasonal surface water collections that are more common close to the lake. Although it is difficult to single out the predominant factors causing the observed difference between BK and the neighboring low-prevalence site Katumba-Songwe, we presume that the difference in altitude and distance to the lake, and their impact on surface water collections, are the most important reasons.

According to our results, RVFV seropositivity seems to be associated with an optimum temperature range. An adverse effect of low temperatures has been shown for RVFV replication and infectiousness, e.g. in the vector *Culex pipiens* [28], [29], [30], while higher temperatures above 27–32°C adversely affect hatching success and size of adult *Aedes aegypti* mosquitoes [31]. The correlation with EVI may be explained by dense vegetation protecting water pools from being heated in the sunlight, and from cooling off at night. Furthermore, vegetation density can be regarded as a proxy for the presence of water. Seasonal increases in vegetation are associated with RVF outbreaks on a larger scale and are used for predicition [32], [33]. Our results confirm this association on a small scale.

It is a limitation of this study that only serologic findings were available for analysis, specific questioning regarding RVF-related symptoms and sequelae to assess clinical significance of serological findings was not possible because samples were analysed retrospectively. However, conduct of this study within the very well characterised EMINI cohort allowed for a detailed analysis of socio-economic, spatial and ecological covariates on a small scale. Given the persistence of IgG responses over several years, the actual date of infection cannot be deduced from these examinations, and socio-economic and environmental conditions at the time of infections may have differed from the time of participant assessment for EMINI. The presence of RVFV IgG in the younger age groups suggests an ongoing or recent virus circulation.

### Public health significance

There are no previous reports of RVF in Mbeya region, and to our knowledge the disease was never diagnosed clinically in Kyela. Since no virus isolation has yet been done in our study, it remains to be elucidated whether the cycling virus is a less virulent RVFV strain such as the apathogenic “clone 13” from the Central African Republic [34], [35], or whether acute cases of RVF have been overlooked in the past. Taking into account the relatively high prevalence for malaria and HIV in the area [36], RVFV encephalitis, retinitis and hemorrhagic fever would be comparatively rare events, which may have been misdiagnosed as malaria- or HIV related morbidity, as is often the case with febrile illnesses in malaria-endemic areas [37], [38].

### Conclusion

In conclusion, this study finds a relatively high RVFV IgG prevalence in an area without previous reports of RVF, and identifies several environmental factors that are associated with RVF infection, independently of age and socio-economic status.

If confirmed in future studies, these findings have important implications in the areas close to Lake Malawi, where health facilities and their staff should be made aware of RVF as a possible diagnosis for their patients. The environmental risk-factors for RVF infection that we identified could serve to predict areas of RVFV endemicity, in addition to outbreak prediction which can be done based on rainfall and vegetation data. It would be interesting to do further studies in similar high risk areas, since it is likely that undetected endemic cycling of RVFV is occurring in many areas apart from our study site.

## Supporting Information

Checklist S1 **STROBE checklist.**
(DOC)

## References

[1] Andriamandimby SF, Randrianarivo-Solofoniaina AE, Jeanmaire EM, Ravololomanana L, Razafimanantsoa LT (2010). Rift Valley fever during rainy seasons, Madagascar, 2008 and 2009.. Emerg Infect Dis.

[2] Jupp PG, Kemp A, Grobbelaar A, Lema P, Burt FJ (2002). The 2000 epidemic of Rift Valley fever in Saudi Arabia: mosquito vector studies.. Med Vet Entomol.

[3] Madani TA, Al-Mazrou YY, Al-Jeffri MH, Mishkhas AA, Al-Rabeah AM (2003). Rift Valley fever epidemic in Saudi Arabia: epidemiological, clinical, and laboratory characteristics.. Clin Infect Dis.

[4] Pepin M, Bouloy M, Bird BH, Kemp A, Paweska J (2010). Rift Valley fever virus(Bunyaviridae: Phlebovirus): an update on pathogenesis, molecular epidemiology, vectors, diagnostics and prevention.. Vet Res.

[5] Sissoko D, Giry C, Gabrie P, Tarantola A, Pettinelli F (2009). Rift Valley fever, Mayotte, 2007–2008.. Emerg Infect Dis.

[6] Fontenille D, Traore-Lamizana M, Zeller H, Mondo M, Diallo M (1995). Short report: Rift Valley fever in western Africa: isolations from Aedes mosquitoes during an interepizootic period.. Am J Trop Med Hyg.

[7] LaBeaud AD, Muchiri EM, Ndzovu M, Mwanje MT, Muiruri S (2008). Interepidemic Rift Valley fever virus seropositivity, northeastern Kenya.. Emerg Infect Dis.

[8] Sang R, Kioko E, Lutomiah J, Warigia M, Ochieng C (2010). Rift Valley fever virus epidemic in Kenya, 2006/2007: the entomologic investigations.. Am J Trop Med Hyg.

[9] Easterday BC, Mc GM, Rooney JR, Murphy LC (1962). The pathogenesis of Rift Valley fever in lambs.. Am J Vet Res.

[10] Turell MJ, Wilson WC, Bennett KE (2010). Potential for North American mosquitoes (Diptera: Culicidae) to transmit rift valley fever virus.. J Med Entomol.

[11] Linthicum KJ, Davies FG, Kairo A, Bailey CL (1985). Rift Valley fever virus (family Bunyaviridae, genus Phlebovirus). Isolations from Diptera collected during an inter-epizootic period in Kenya.. J Hyg (Lond).

[12] Anyamba A, Chretien JP, Small J, Tucker CJ, Formenty PB (2009). Prediction of a Rift Valley fever outbreak.. Proc Natl Acad Sci U S A.

[13] Pourrut X, Nkoghe D, Souris M, Paupy C, Paweska J (2010). Rift Valley fever virus seroprevalence in human rural populations of Gabon.. PLoS Negl Trop Dis.

[14] Kolenikov S, Angeles G (2009). Socioeconomic status measurment with discrete poxy variables: is principal component analysis a reliable answer?. Review of Income and Wealth.

[15] Vyas S, Kumaranayake L (2006). Constructing socio-economic status indices: how to use principal components analysis.. Health Policy Plan.

[16] Filmer D, Pritchett LH (2001). Estimating wealth effects without expenditure data–or tears: an application to educational enrollments in states of India.. Demography.

[17] Farr TG, Rosen PA, Caro E, Crippen R, Duren R (2007). The Shuttle Radar Topography Mission.. Rev Geophys.

[18] Rodríguez E, Morris CS, Belz JE, Chapin EC, Martin JM (2005). An Assessment of the SRTM Topographic Products.

[19] Wang Z, Wang P, Li Z (2004). Using MODIS Land Surface Temperature and Normalized Difference Vegetation Index products for monitoring drought in the southern Great Plains, USA.. International Journal of Remote Sensing.

[20] Anonymous (2008). MODIS Reprojection Tool User's Manual, Release 4.0..

[21] Swanepoel R, Struthers JK, Erasmus MJ, Shepherd SP, McGillivray GM (1986). Comparison of techniques for demonstrating antibodies to Rift Valley fever virus.. J Hyg (Lond).

[22] Barros AJ, Hirakata VN (2003). Alternatives for logistic regression in cross-sectional studies: an empirical comparison of models that directly estimate the prevalence ratio.. BMC Med Res Methodol.

[23] Spiegelman D, Hertzmark E (2005). Easy SAS calculations for risk or prevalence ratios and differences.. Am J Epidemiol.

[24] Kirby MJ, West P, Green C, Jasseh M, Lindsay SW (2008). Risk factors for house-entry by culicine mosquitoes in a rural town and satellite villages in The Gambia.. Parasit Vectors.

[25] Clements AC, Pfeiffer DU, Martin V, Pittliglio C, Best N (2007). Spatial risk assessment of Rift Valley fever in Senegal.. Vector Borne Zoonotic Dis.

[26] Nguku PM, Sharif SK, Mutonga D, Amwayi S, Omolo J (2010). An investigation of a major outbreak of Rift Valley fever in Kenya: 2006–2007.. Am J Trop Med Hyg.

[27] Loscher T, Burchard G-D (2010). Tropenmedizin in Klinik und Praxis. 4 ed.

[28] Brubaker JF, Turell MJ (1998). Effect of environmental temperature on the susceptibility of Culex pipiens (Diptera: Culicidae) to Rift Valley fever virus.. J Med Entomol.

[29] Turell MJ (1993). Effect of environmental temperature on the vector competence of Aedes taeniorhynchus for Rift Valley fever and Venezuelan equine encephalitis viruses.. Am J Trop Med Hyg.

[30] Turell MJ, Rossi CA, Bailey CL (1985). Effect of extrinsic incubation temperature on the ability of Aedes taeniorhynchus and Culex pipiens to transmit Rift Valley fever virus.. Am J Trop Med Hyg.

[31] Mohammed A, Chadee DD (2011). Effects of different temperature regimens on the development of Aedes aegypti (L.) (Diptera: Culicidae) mosquitoes.. Acta Trop.

[32] Anyamba A, Linthicum KJ, Small J, Britch SC, Pak E (2010). Prediction, assessment of the Rift Valley fever activity in East and Southern Africa 2006–2008 and possible vector control strategies.. Am J Trop Med Hyg.

[33] Abdo-Salem S, Tran A, Grosbois V, Gerbier G, Al-Qadasi M (2011). Can Environmental and Socioeconomic Factors Explain the Recent Emergence of Rift Valley Fever in Yemen, 2000–2001?. Vector Borne Zoonotic Dis.

[34] Muller R, Saluzzo JF, Lopez N, Dreier T, Turell M (1995). Characterization of clone 13, a naturally attenuated avirulent isolate of Rift Valley fever virus, which is altered in the small segment.. Am J Trop Med Hyg.

[35] Sall AA, de AZPM, Zeller HG, Digoutte JP, Thiongane Y (1997). Variability of the NS(S) protein among Rift Valley fever virus isolates.. J Gen Virol.

[36] Jordan-Harder B, Maboko L, Mmbando D, Riedner G, Nagele E (2004). Thirteen years HIV-1 sentinel surveillance and indicators for behavioural change suggest impact of programme activities in south-west Tanzania.. AIDS.

[37] Chandler CI, Chonya S, Boniface G, Juma K, Reyburn H (2008). The importance of context in malaria diagnosis and treatment decisions - a quantitative analysis of observed clinical encounters in Tanzania.. Trop Med Int Health.

[38] Koram KA, Molyneux ME (2007). When is “malaria” malaria? The different burdens of malaria infection, malaria disease, and malaria-like illnesses.. Am J Trop Med Hyg.

