# Supplementary material for: High Seroprevalence of Rift Valley Fever and Evidence for Endemic Circulation in Mbeya Region, Tanzania, in a Cross-Sectional Study
Source: PLoS Negl Trop Dis. 2012 Mar 27;6(3):e1557. doi: 10.1371/journal.pntd.0001557 (PMC3313937; doi:10.1371/journal.pntd.0001557)
Supplement: Checklist S1 — STROBE checklist. (DOC) [file pntd.0001557.s001.doc]

STROBE Statement—checklist of items that should be included in reports of observational studies

|  | Item No | Recommendation |
| --- | --- | --- |
| **Title and abstract** | 1 | (*a*) Indicate the study’s design with a commonly used term in the title or the abstract Fulfilled through a change in title |
|  |  | (*b*) Provide in the abstract an informative and balanced summary of what was done and what was found  Fulfilled (Abstract) |
| Introduction | | |
| Background/rationale | 2 | Explain the scientific background and rationale for the investigation being reported  Fulfilled (Introduction) |
| Objectives | 3 | State specific objectives, including any prespecified hypotheses  Fulfilled (Introduction) |
| Methods | | |
| Study design | 4 | Present key elements of study design early in the paper  Fulfilled (Introduction) |
| Setting | 5 | Describe the setting, locations, and relevant dates, including periods of recruitment, exposure, follow-up, and data collection  Fulfilled (Methods) |
| Participants | 6 | (*a*) *Cohort study*—Give the eligibility criteria, and the sources and methods of selection of participants. Describe methods of follow-up Not applicable (N/A)  *Case-control study*—Give the eligibility criteria, and the sources and methods of case ascertainment and control selection. Give the rationale for the choice of cases and controls N/A  *Cross-sectional study*—Give the eligibility criteria, and the sources and methods of selection of participants Fulfilled (Methods) |
|  |  | (*b*) *Cohort study*—For matched studies, give matching criteria and number of exposed and unexposed N/A  *Case-control study*—For matched studies, give matching criteria and the number of controls per case N/A |
| Variables | 7 | Clearly define all outcomes, exposures, predictors, potential confounders, and effect modifiers. Give diagnostic criteria, if applicable Fulfilled (Results) |
| Data sources/ measurement | 8* | For each variable of interest, give sources of data and details of methods of assessment (measurement). Describe comparability of assessment methods if there is more than one group Fulfilled (Methods) |
| Bias | 9 | Describe any efforts to address potential sources of bias Fulfilled (Methods) |
| Study size | 10 | Explain how the study size was arrived at Fulfilled (Introduction, Methods) |
| Quantitative variables | 11 | Explain how quantitative variables were handled in the analyses. If applicable, describe which groupings were chosen and why Fulfilled (Methods) |
| Statistical methods | 12 | (*a*) Describe all statistical methods, including those used to control for confounding Fulfilled (Methods, Results) |
|  |  | (*b*) Describe any methods used to examine subgroups and interactions Fulfilled (Methods, Results) |
|  |  | (*c*) Explain how missing data were addressed Fulfilled (Methods) |
|  |  | (*d*) *Cohort study*—If applicable, explain how loss to follow-up was addressed N/A  *Case-control study*—If applicable, explain how matching of cases and controls was addressed N/A  *Cross-sectional study*—If applicable, describe analytical methods taking account of sampling strategy Fulfilled (Methods) |
|  |  | (*e*) Describe any sensitivity analyses Fulfilled (Methods) |

Continued on next page

| Results | | |
| --- | --- | --- |
| Participants | 13* | (a) Report numbers of individuals at each stage of study—eg numbers potentially eligible, examined for eligibility, confirmed eligible, included in the study, completing follow-up, and analysed Fulfilled (Methods, Results) |
|  |  | (b) Give reasons for non-participation at each stage N/A |
|  |  | (c) Consider use of a flow diagram N/A |
| Descriptive data | 14* | (a) Give characteristics of study participants (eg demographic, clinical, social) and information on exposures and potential confounders Fulfilled (Results, Table 1) |
|  |  | (b) Indicate number of participants with missing data for each variable of interest Fulfilled (Results) |
|  |  | (c) *Cohort study*—Summarise follow-up time (eg, average and total amount) N/A |
| Outcome data | 15* | *Cohort study*—Report numbers of outcome events or summary measures over time N/A |
|  |  | *Case-control study—*Report numbers in each exposure category, or summary measures of exposure N/A |
|  |  | *Cross-sectional study—*Report numbers of outcome events or summary measures Fulfilled (Results) |
| Main results | 16 | (*a*) Give unadjusted estimates and, if applicable, confounder-adjusted estimates and their precision (eg, 95% confidence interval). Make clear which confounders were adjusted for and why they were included Fulfilled (Results, Tables 2, 3) |
|  |  | (*b*) Report category boundaries when continuous variables were categorized N/A |
|  |  | (*c*) If relevant, consider translating estimates of relative risk into absolute risk for a meaningful time period N/A |
| Other analyses | 17 | Report other analyses done—eg analyses of subgroups and interactions, and sensitivity analyses Fulfilled (Results) |
| Discussion | | |
| Key results | 18 | Summarise key results with reference to study objectives Fulfilled (Discussion) |
| Limitations | 19 | Discuss limitations of the study, taking into account sources of potential bias or imprecision. Discuss both direction and magnitude of any potential bias Fulfilled (Discussion) |
| Interpretation | 20 | Give a cautious overall interpretation of results considering objectives, limitations, multiplicity of analyses, results from similar studies, and other relevant evidence Fulfilled (Discussion) |
| Generalisability | 21 | Discuss the generalisability (external validity) of the study results Fulfilled (Discussion, Conclusion) |
| Other information | | |
| Funding | 22 | Give the source of funding and the role of the funders for the present study and, if applicable, for the original study on which the present article is based Fulfilled (financial disclosure section) |

*Give information separately for cases and controls in case-control studies and, if applicable, for exposed and unexposed groups in cohort and cross-sectional studies.

**Note:** An Explanation and Elaboration article discusses each checklist item and gives methodological background and published examples of transparent reporting. The STROBE checklist is best used in conjunction with this article (freely available on the Web sites of PLoS Medicine at http://www.plosmedicine.org/, Annals of Internal Medicine at http://www.annals.org/, and Epidemiology at http://www.epidem.com/). Information on the STROBE Initiative is available at www.strobe-statement.org.
